# High Connectivity of the White Seabream (*Diplodus sargus*, L. 1758) in the Aegean Sea, Eastern Mediterranean Basin

**DOI:** 10.3390/ani9110979

**Published:** 2019-11-15

**Authors:** Athanasios Exadactylos, Dimitrios Vafidis, Costas S. Tsigenopoulos, Georgios A. Gkafas

**Affiliations:** 1 Department of Ichthyology and Aquatic Environment, School of Agriculture Sciences, University of Thessaly, 38446 Volos, Greece; dvafidis@uth.gr; 2 Institute of Marine Biology, Biotechnology and Aquaculture, Hellenic Centre for Marine Research, 71003 Heraklion, Greece; tsigeno@hcmr.gr

**Keywords:** connectivity, Mediterranean, population structure, microsatellites

## Abstract

Population dynamics in the marine realm can shape species’ spatial structure and genetic variability between given geographical areas. Connectivity is an important factor of species’ population structure. In this study, we examined the genetic diversity and structure of white seabream (*Diplodus sargus*, L. 1758) in the eastern Mediterranean basin, using a panel of four microsatellite markers. Recorded low *F_ST_* values within the study area indicate little evidence of genetic differentiation among populations. Results suggest high gene flow which may imply near-panmixia between populations, indicating the possibility of a probable movement of adult migrants, or strong passive drift at sea in early life stages of the species. To this extent, bibliographically speaking, different species within the Sparidae family favor altered population dynamics patterns with respect to local populations and genetic divergence, in the context of the molecular marker used.

## 1. Introduction

Population structure at sea is cryptic due to the absence of physical barriers. However, with respect to different marine taxa, population structure can differ, e.g., [1,2] depending on life history traits, and it can be used to better understand population connectivity and population dynamics [3]. The white seabream (*Diplodus sargus*, L. 1758) populations favor a typical homogeneous mixture of individuals, thus suggesting low genetic differentiation and high levels of connectivity [4,5,6]. However, sister species within the Sparidae family seem to follow a differential strategy; such is the case of saddled seabream (*Oblada melanura*, L. 1758), where statistically significant genetic differentiation among populations in the Aegean Sea illustrates a potential small-scale population structure [7]. On the other hand, connectivity in the marine environment is a rather common situation due to potential complex migratory patterns and admixture; under the population genetic framework one could evaluate the scale of dispersal of marine taxa [8]. Indeed, documented species with long-lived larval stages favor relatively low population structure and high gene flow and theoretically can enable connectivity over large distances due to life-history traits and environmental pressures [9]. Also expected is that long-lived larvae under passive drift form panmictic stocks [10] and dispersal is likely to occur mainly during the pelagic larval phase before settlement [11]. However, massive larvae dispersal by hydrographical processes is very unlikely to occur [12]. Barcelloni et al. [13] suggest that ecological/historical factors might have caused discrepancy in the geographical distribution of genetic variation among otherwise biologically similar species.

Inferring demographic connectivity from molecular markers has gained awareness, especially for conservation and fisheries management purposes [14]. Fisheries management modeling can provide a holistic view of stocks’ dynamics giving a better understanding on structure and connectivity [15]. Ecological management modeling can play a vital role in improving complex population structure within certain areas, shedding light on stock identification [16].

These two features, a focus on larval survivors and an ability to examine long-term mean population connectivities, are critical contributions that genetic studies can make to marine population dynamics. However, genetic surveys of marine populations also face a number of severe challenges that have limited the impact of these approaches on marine ecology, coastal management, or fisheries preservation. The challenges inherent in such accurate genetic determinations have slowed the use of genetic data to estimate real-time population connectivities and have frustrated the use of genetics in many conservation and fisheries contexts [14].

Neutral markers, such as microsatellites, have been used exclusively to identify the geographic structure of subpopulations and estimate the genetic connectivity. In this study, four microsatellites were used to assess the population structure of the white seabream. The white seabream belongs to the Sparidae family and it is found from the Atlantic to the Indian Ocean, in the Mediterranean Sea and the Persian Gulf [17]. Pelagic larvae behavioral pattern and juvenile migration movement towards deeper habitats have been illustrated so far [18], in order to discuss the species recruitment strategy into adult populations. Here, we addressed the level of genetic diversity and discussed the genetic structure strategy of the species in question in the eastern Mediterranean basin.

## 2. Material and Methods

### 2.1. Sampling Design and Molecular Techniques

**Figure 1:**
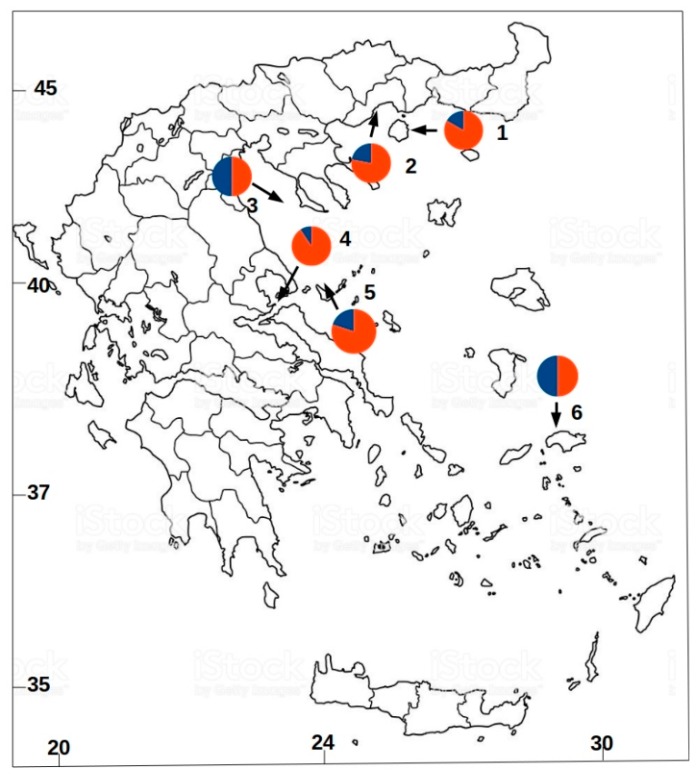
Sampling locations in the Aegean Sea and structure pies with locprior K = 2 (different colors represent the two different genetic clusters). Numbers indicate the sampled area of individuals. 1: Thasos (THA)—30 specimens; 2: Kavala (KAV)—24 specimens; 3: Chalkidiki (CHA)—28 specimens; 4: Trikeri (TRI)—28 specimens; 5: Sporades (SPO)—26 specimens; 6: Samos (SAM)—30 specimens.

Samples from local fish markets were collected from six different geographical areas in the Aegean Sea, as shown in Figure 1. The total sample size was 166 mature specimens. An adequate proportion of muscle tissues were stored in 20% DMSO NaCl 5M. DNA was extracted following the standard phenol/chloroform extraction protocol [19]. DNA was preserved in 10 mM TE (Tris-HCl, EDTA) and stored in −20 °C. A total panel of four DNA microsatellite markers [20,21] were tested and optimized for the genetic analyses (Table 1) due to their high variability (see review in [20,21]). A multiplex PCR Kit (Qiagen) with a hot start Taq polymerase was used for the DNA amplifications. The PCR cycling profile was: 95 °C for 15 min; 30 cycles of 95 °C for 1 min, annealing temperature (Table 1) for 30 s and 72 °C for 30 s; 72 °C for 15 min. PCR products were verified by agarose gel electrophoresis. Amplified DNA products were screened on an ABI 3730 DNA Analyzer (Applied Biosystems). Each specimen’s alleles were scored by the STRand software v.2.0 [22] and the 10% of genotypes were re-assessed for error checking.

### 2.2. Statistics

All loci were tested for the presence of null alleles, or allelic dropout using the software Micro-Checker v. 2.2.3 [23], where a Monte Carlo simulation method was used to generate expected homozygote and heterozygote allele size difference frequencies. Exact tests for Hardy–Weinberg equilibrium, linkage disequilibrium (using Fisher’s Exact Test), expected heterozygosity (*H_EXP_*) and observed heterozygosity (*H_OBS_*) were carried out using the software Genepop v. 3.4 [24]. Fixation indexes *F_ST_* and *F_IS_* (using the formulations described by [25]), number of alleles per locus were calculated using the FSTAT v. 2.9.3.2 software [26]. Population structure was further assessed using the software LEA as implemented in R platform [27] to test for presence of distinct genetic clusters and subpopulations. The admixture model-based STRUCTURE simulations were conducted at varying levels of possible population numbers. To test the convergence of the priors and the appropriateness of the chosen burn-in length and simulation length, three independent repeats were run for each value of K (1 ≤ K ≤ 10). Burn-in length and length of simulation were set at 500,000 and 1,000,000 repetitions, respectively. The cross-entropy criterion was used for choosing the number of genetic clusters. This criterion is based on the prediction of a fraction of masked genotypes (matrix completion), and on the cross-validation approach. Smaller values of the cross-entropy criterion mean better runs. Significance level was adjusted according to Bonferroni correction [28].

## 3. Results

**Figure 2:**
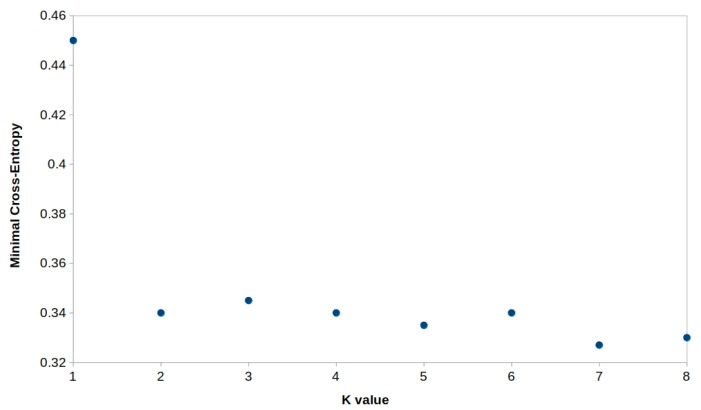
Cross-entropy plot for the number of cluster K = 1–8. The retained value of K is K = 2.

Among the four loci screened (a subset of the genetic data can be found in the Supplementary Materials Table S1), none of them showed evidence of null alleles. The observed heterozygosity values were high enough (0.580 ± 0.156 s.d. to 0.917 ± 0.096 s.d.), but within the range observed in the bibliography for fish species. Nevertheless, no statistical departure from the Hardy–Weinberg law was detected (*P_99_* criterion), as shown in Table 1. The mean genetic heterogeneity value (*F_ST_*) was quite low (0.008 ± 0.010 s.d.) indicating little evidence of genetic differentiation among populations (Table 2). The number of genetic clusters through LEA program was based on the cross-entropy criterion (Figure 2), revealing the presence of two subpopulations, which also indicates high gene flow and admixture of individuals among the studied geographical area (Figure 1).

## 4. Discussion

The present study indicated a significant connectivity pattern for contiguous populations of white seabream throughout the study area. The structural analysis result was that of a two cluster assignment of populations which significantly correspond to high mixing of all individuals. Published studies show similar a genetic homogeneity strategy for the species in question within the Mediterranean Sea, e.g., see [4,5]. The Aegean Sea exhibits genetic continuity of white seabream populations, which may be influenced by the ocean currents, in the context of the relatively long planktonic larval phases that suggest relatively high connectivity [29] and possible differential recruitment process [6].

On the other hand, according to previously reported studies of other benthopelagic Sparidae species [7], north and central Aegean populations seem to force species to form and exhibit complex spatial patterns with respect to their benthic and geographic abundance [30]. This might be explained by the dynamic status of the eastern Mediterranean Sea with respect to the physiographic and hydrodynamic complexity, such as the prevailing unique oceanographic features and different water masses compared to the western Mediterranean basin; see review in [31]. Previous studies within the Aegean Sea show absence of interpopulation genetic structure [32], suggesting the existence of single stocks within certain areas within the eastern Mediterranean basin [33]. This might be due to large effective population sizes that limit genetic drift and life history characteristics that favor dispersal in continuous dynamic oceanic environments (see review in [34]).

Taking into consideration the results presented here, we suggest a considerably high connectivity for this Sparidae species in the eastern Mediterranean Sea. This may be an effect of the mixing of adult individuals in apparently similar geographic areas, which may also contribute to the low differentiation and high connectivity between samples within the Aegean Sea. Different strategies within Sparidae family, with respect to different larvae phases, seem to shape differential genetic composition and population dynamics, e.g., see [8]. Moreover, oceanographic and environmental features affect larval behavior, forcing structuring mechanisms to dispersal, retention or larvae settlement [35]. This larval drift may shape population differentiation and is considered of high importance of connectivity [36]. Seasonal shifts in habitat uses, life cycle, and, most important, spawning fidelity are a common strategy of many species [37]. Also, species’ phenotypic plasticity and diet behavior influence different feeding strategies, resulting altered ecological behaviors [38]. Such an ecological alteration is often characterized by strong seasonality through annual temporal patterns of biological processes [39]. It is then suggested that species-specific temporal patterns were found demonstrating a clear annual temporal niche partitioning within the Sparidae family [40].

However, one should take into account that sample sizes and the panel of microsatellite markers are just on the verge of the scientific protocol adequacy. Species’ geographical dispersal might be closely linked to oceanographic features and specific ecological habitats and can provide an efficient tool for distribution and abundance identification of all Sparidae species in a conservation management plan for the eastern Mediterranean basin. Populations of the same species may vary in experiencing different recruitment success or survival rates under different environmental conditions [16,41]. The complex nature of a population structure may mislead the spatially sampled stock that is used for management purposes [42]. One such example is the collapse of Atlantic cod, due to the ineffective recognition to acknowledge stock boundaries, complex structure, and spawning areas in the north Atlantic [43,44,45]. Thus, a better understanding of spatial structure and connectivity may play a key role with respect to fisheries management such as recruitment, overfishing, conservation, and environmental interactions [46].

These findings reveal complex population dynamics patterns within the Sparidae family and therefore have important implications on differential policies for the effective conservation and management for each species within the family. 

## Figures and Tables

**Table 1:** The panel of the four microsatellite DNA markers with annealing temperature in parentheses. NoA: number of alleles; *H_EXP_*: expected heterozygosity; *H_OBS_*: observed heterozygosity.

Microsatellite DNA Markers	Genetic Indices	TRI	SPO	THA	KAV	CHA	SAM
*Pma1* (57 °C)	NoA	10	15	14	13	19	7
	*H_EXP_*	0.970	0.974	0.949	0.921	0.957	0.909
	*H_OBS_*	1.000	0.900	0.667	0.714	0.833	0.833
*Pma2* (57 °C)	NoA	9	11	10	13	17	9
	*H_EXP_*	0.955	0.895	0.866	0.812	0.924	0.955
	*H_OBS_*	1.000	0.900	0.917	0.714	0.833	1.000
*SAI10* (58 °C)	NoA	9	11	11	12	18	11
	*H_EXP_*	0.955	0.932	0.917	0.910	0.952	0.985
	*H_OBS_*	0.667	0.700	0.500	0.428	0.647	1.000
*SAI12* (59 °C)	NoA	8	10	12	14	16	9
	*H_EXP_*	0.939	0.889	0.942	0.951	0.928	0.955
	*H_OBS_*	0.500	0.500	0.583	0.462	0.667	0.833

**Table 2:** *F_ST_* pairwise values of the white seabream populations within the Aegean Sea.

Populations	TRI	SPO	THA	KAV	CHA	SAM
SPO	−0.0074	0				
THA	−0.0111	−0.0006	0			
KAV	−0.0148	0.0039	0.0096	0		
CHA	−0.0057	0.0192	0.0053	0.0151	0	
SAM	0.0047	0.0105	0.0213	0.0169	0.0060	0

## Supplementary Materials

The following are available online at https://www.mdpi.com/2076-2615/9/11/979/s1, Table S1: Subset of the microsatellite genetic data.
