# Addendum: Exadactylos, A., et al. High Connectivity of the White Seabream (*Diplodus sargus*, L. 1758) in the Aegean Sea, Eastern Mediterranean Basin. *Animals* 2019, *11*, 979

**DOI:** 10.3390/ani9121152

**Published:** 2019-12-16

**Authors:** Athanasios Exadactylos, Dimitrios Vafidis, Costas S. Tsigenopoulos, Georgios A. Gkafas

**Affiliations:** 1 Department of Ichthyology and Aquatic Environment, School of Agriculture Sciences, University of Thessaly, 38446 Volos, Greece; dvafidis@uth.gr; 2 Institute of Marine Biology, Biotechnology and Aquaculture, Hellenic Centre for Marine Research, 71003 Heraklion, Greece; tsigeno@hcmr.gr

The authors wish to make the following corrections to this paper [1]:

Add the simple summary:

**Simple summary:** Population dynamics is important for the estimation of the ecology and conservation status of the species in question. Levels of genetic structure, connectivity, gene flow and genetic diversity often differ in the marine environment due to the apparent lack of barriers. In the present study, the population structure of a highly economical species, the white seabream, was evaluated using short tandem repeats. Results suggest high connectivity in the Aegean sea, eastern Mediterranean sea, indicating the possibility of a probable movement of adult migrants or strong passive drift at sea in the early life stages of the species. However, it is clear that different species within the Sparidae family favor altered strategies, as discussed in the study, and information of this kind needs to be evaluated by ecologists with respect to potential different conservation and resources-management policies for the eastern Mediterranean basin.

## References

[1] Exadactylos A., Vafidis D., Tsigenopoulos C.S., Gkafas G.A. (2019). High Connectivity of the White Seabream (*Diplodus sargus*, L. 1758) in the Aegean Sea, Eastern Mediterranean Basin. Animals.

